# Urinoma Masquerading as Post-hysterectomy Hematoma: The Role of Accurate Diagnosis and Imaging-Guided Intervention

**DOI:** 10.7759/cureus.63235

**Published:** 2024-06-26

**Authors:** Sufia Athar, Saheed A Shittu, Asim Abduh A Alhattami, Sahar Fatima, Kholode Khalid Al-Maslamani, Lolwa Alansari

**Affiliations:** 1 Obstetrics and Gynaecology, Al Wakra Hospital, Hamad Medical Corporation, Doha, QAT; 2 Obstetrics and Gynaecology, Hamad Medical Corporation, Doha, QAT; 3 Urology, Hamad Medical Corporation, Al Wakra, QAT; 4 Radiology, Hamad Medical Corporation, Al Wakra, QAT; 5 Obstetrics and Gynaecology, Hamad Medical Corporation, Al Wakra, QAT

**Keywords:** complication, computed tomography, pelvic collection, hysterectomy, ureteric injury

## Abstract

Although rare, ureteric injuries can occur during gynecological surgical interventions, and their diagnosis can be challenging, especially when delayed. If left untreated, missed ureteric injuries can lead to severe complications, including prolonged hospitalization, sepsis, renal damage, and potentially even loss of the affected kidney. We present a unique case of a urinoma caused by bilateral ureteric injuries following abdominal hysterectomy, which was initially misdiagnosed as an intraperitoneal hematoma. However, further radiological investigations enabled accurate diagnosis without the need for exploratory laparotomy, demonstrating the importance of thorough evaluation for all possible complications in patients presenting with post-operative issues. In cases of pelvic collections of unclear origin or ureteric injury, a computed tomography (CT) scan is the gold standard diagnostic modality, providing precise diagnosis and expedited management.

## Introduction

Hysterectomy represents the most common non-pregnancy-related major operation performed on women in the United States, with approximately over 500,000 done yearly for a variety of mostly benign disorders affecting the female genital tract. It can be performed via an open, laparoscopic, or vaginal approach. Worldwide, the commonest route for hysterectomy has, over the past decade, become laparoscopic. Hysterectomy is associated with morbidity and occasional mortality. Iatrogenic complications represent an increasing concern for gynecologists [1].

The risk of iatrogenic injury to the bladder or ureters during a hysterectomy is relatively low, ranging from 0.21% to 1.5% [1,2]. The likelihood of injury varies depending on the surgical approach. According to the available data, the incidence of ureteric injury is highest during a total laparoscopic hysterectomy, at 0.31%. This is followed by laparoscopic-assisted vaginal hysterectomy (0.29%) and total vaginal hysterectomy (0.24%) as the second and third most common causes, respectively. Total abdominal hysterectomy accounts for 0.2% of these cases. The lowest incidence of ureteric injury is observed during a laparoscopic subtotal hysterectomy, at 0.14%. However, the likelihood of injury varies depending on the surgical approach, with the highest incidence observed during a total laparoscopic hysterectomy and the lowest incidence observed during a laparoscopic subtotal hysterectomy [1,2].

While ureteric injuries are relatively rare, if left untreated, they can have severe and far-reaching consequences, including prolonged hospitalization, intra-abdominal sepsis, renal impairment or failure, and the need for repeated surgeries or interventional procedures. In addition, patients may experience prolonged recovery times, loss of employment, and even disability. The financial and emotional toll of these complications can be significant, especially if the injury is not detected immediately. Furthermore, the risk of litigation and ongoing medical care can also have a lasting impact on patients. It is essential that healthcare providers are aware of the potential risks associated with ureteric injuries and take proactive measures to prevent and detect them promptly. By doing so, patients can receive timely and effective treatment, minimizing the risk of long-term consequences and improving overall outcomes [3].

Urinoma is a mass of encapsulated urine collection due to continuous extravasation due to interruption in the urinary collection system, producing internalized urine. It may occur spontaneously in the presence of obstruction after a closed renal or urinary injury or surgical operation [4].

While post-traumatic urinary incontinence is a relatively common occurrence, ranging from 2% to 18% [1], the development of a urinoma is a rare and distinct condition. In fact, only a few cases have been reported in the medical literature. In some cases, a urinoma may not cause symptoms initially but can later lead to delayed complications, such as electrolyte imbalances, hydronephrosis, and paralytic ileus [1-3]. This highlights the importance of prompt recognition and management of this condition to prevent or mitigate its potential consequences. If mixed with blood, they may be diagnosed as hematoma. Computed tomography (CT) remains the gold standard for diagnosing post-operative ureteric injury, and it not only differentiates between the different causes of the pelvic collection but also indicates the site of the ureteric injury [2,3,5].

We report a rare case where a urinoma was developed because of urine leakage from bilateral ureteric injuries that occurred after an abdominal hysterectomy. The urinoma was initially misdiagnosed as an intraperitoneal hematoma. Appropriate radiological investigations enhanced the accurate diagnosis of urinoma and led to avoiding inappropriate exploratory laparotomy.

## Case presentation

First presentation to ED

A 41-year-old woman, P2, presented to the emergency department (ED) for adults with abdominal pain and bloating. She had undergone an abdominal hysterectomy for large fibroids six days earlier in a private hospital. Her post-operative period in the primary hospital was unremarkable, and she was discharged home on the third post-operative day. She had normal bowel and bladder function. Vaginal bleeding was mild. However, from post-operative day 4, she developed pain in the left side of the abdomen, which was associated with distension of the abdomen. She did not have a fever, nausea, vomiting, abnormal vaginal discharge, or urinary symptoms. Her previous medical history was insignificant. She had a prior history of an abdominal myomectomy, which was performed one year ago in the same hospital. She did not smoke and had an unremarkable family history.

Clinical examination

Vital signs were normal on clinical examination (temperature, 37.3°C (oral); heart rate, 89/minute; respiratory rate, 19/minute; blood pressure, 135/89 mmHg; oxygen saturation, 100%). Respiratory, cardiovascular, and neurological exams were unremarkable. On abdominal examination, the wound was well approximated with minimal brownish discharge. On palpation, her abdomen was soft, with mild tenderness and a palpable mass in the lower abdomen. There was no vaginal loss.

Laboratory results and radiological evaluation

**Figure 1:**
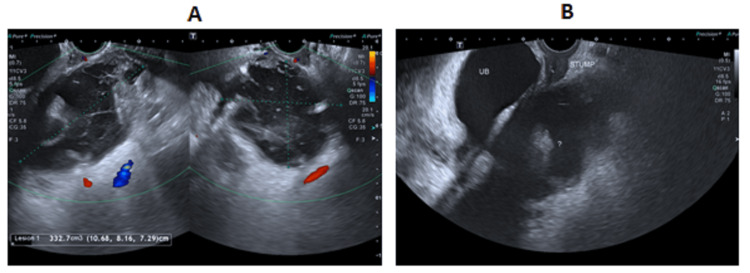
Ultrasound images (first ED visit) (A) Large heterogeneous pelvic collection (hematoma) showing septations and internal echoes. (B) Post-hysterectomy status with the vaginal stump. ED, emergency department

The results of the laboratory tests showed a mild leukocytosis (13.1 × 10^3/uL), with a slight increase in alanine transaminase (ALT = 72 U/L) and aspartate transaminase (AST = 72 U/L). Her hemoglobin was 10 g/dL, C-reactive protein (CRP) was 5 mg/L, and the results of the renal function tests were in the normal range. Ultrasound of the pelvis (Figure 1) was reported as a pelvic collection (hematoma) measuring 10.6 × 8.1 × 7.3 cm, volume 332 mL with septations and echogenic components. As she was clinically stable, a diagnosis of post-hysterectomy hematoma was made. Broad-spectrum antibiotics, analgesics, and follow-up were requested. She was discharged from the ED in stable condition.

Second presentation to ED

She reported back after four days (post-operative day 10) again with abdominal pain, progressive distention, and frequent vomiting. She did not report any symptoms of fever, unusual vaginal discharge, or urinary issues. On clinical examination, she looked pale and anxious with pain (T, 36.8°C (oral); RR, 18; BP, 157/82; SpO2, 98%). On abdominal examination, the wound was well approximated. On palpation, her abdomen was tense, with generalized abdominal tenderness. There was no vaginal loss.

Laboratory results and radiological evaluation

Laboratory results revealed a decrease in hemoglobin from 10 to 8.8 gm/dL, an increased total leukocyte count of 14.3 × 10^3/uL, and an elevated CRP of 89 mg/L. Serum creatinine was 248 umol/L (normal range, 50-98 umol/L) with an elevated prothrombin time of 18.1 seconds (normal range, 9.4-12.5 s) and an activated partial thromboplastin time of 39.6 s (normal range, 25.1-36.5 s). Liver enzymes were normal.

Diagnosis and challenges in diagnosis

**Figure 2:**
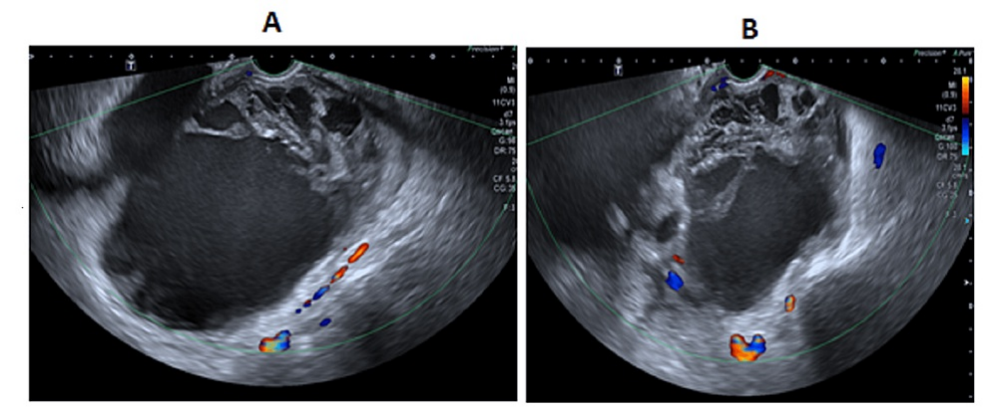
Ultrasound images (second ED visit) A follow-up pelvic scan showing a pelvic collection of 15.3 × 11.7 × 11.9 cm, 1122.3 cc in volume (previously 332 mL). ED, emergency department

**Figure 3:**
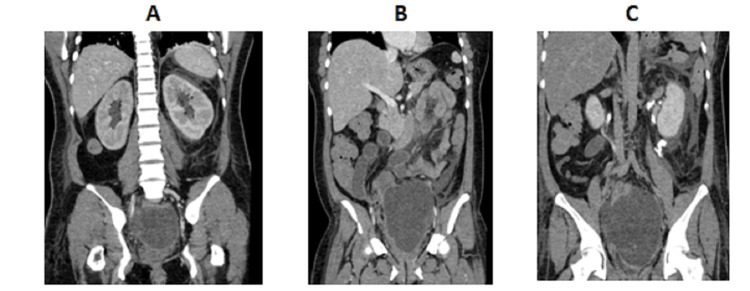
Coronal CT scan with IV contrast (following abnormal results of renal function tests) (A,B) Images showing bilateral renal calyceal dilatation, left perirenal fat stranding, and large hypodense pelvic collection. (C) Delayed images showing contrast extravasation from the left calyceal system and pelvic collection. Following abnormal results on renal function tests, a CT scan was performed to investigate the cause of the altered renal function, which ultimately led to the diagnosis of bilateral ureteric injuries and a urinoma. CT, computed tomography

**Figure 4:**
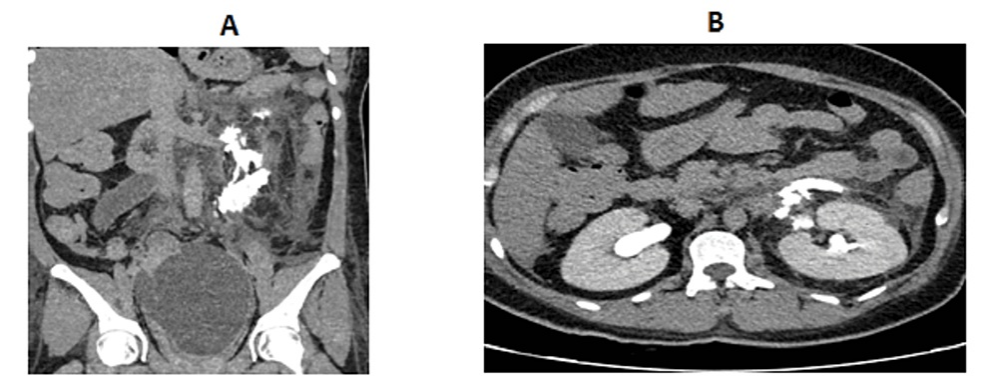
Delayed CT with IV contrast image showing contrast extravasation from the left renal calyceal system due to distal left ureteric obstruction secondary to pelvic collection (A) Coronal CT. (B) Axial CT. CT, computed tomography

**Figure 5:**
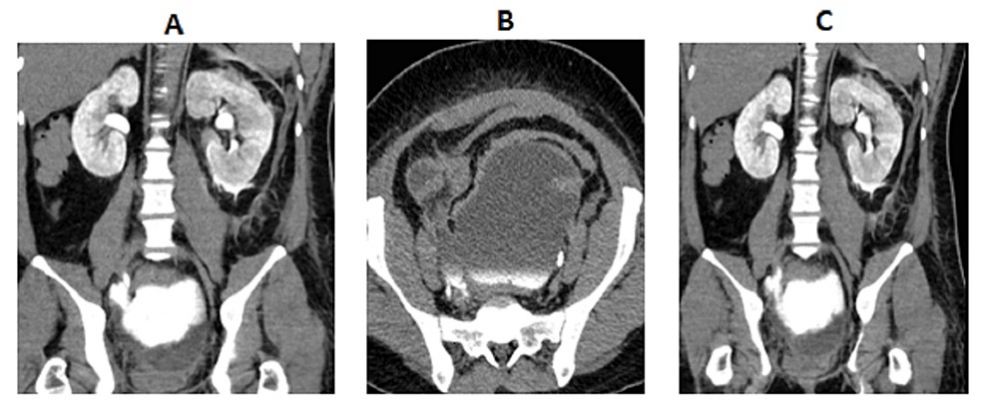
Coronal CT with IV contrast showing right distal ureteric rupture with pooling of contrast from the right side filling up pelvic collection CT, computed tomography

**Figure 6:**
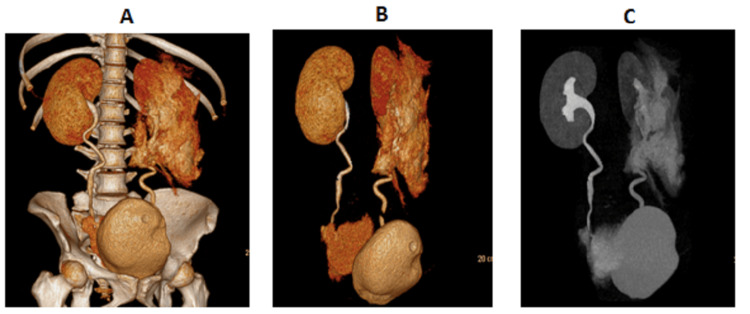
CT urogram (A,B) With contrast: post-processed 3D images further demonstrate right distal ureteric rupture with distal right ureteric extravasation of contrast leading to large pelvic urinoma that was earlier diagnosed as large pelvic collection/hematoma. (C) Without contrast: extravasation from left pelvicalyceal rupture secondary to back pressure from left distal ureteric obstruction. Right distal ureteric rupture with distal right ureteric extravasation of contrast leading to large pelvic urinoma. A CT urogram was performed to further investigate and confirm the diagnosis of ureteric injury following initial CT findings. CT, computed tomography

Based on her clinical findings, laboratory results, and previous sonography results, pelvic hematoma and sepsis were suspected. A repeat ultrasound was performed (Figure 2). An intra-abdominal fluid collection with internal echoes and septa was seen in the pelvic cavity close to the uterine bed, measuring approximately 15.3 × 11.7 × 11.9, 1122.3 cc in volume (previously 332 mL on last sonography, four days back). Bilateral prominent renal pelvic cysts were noted with minimal free fluid in the left upper quadrant and left perinephric area. A laparotomy was planned due to the significant increase in size of the pelvic hematoma. The ureteral injury was suspected due to elevated creatinine and radiological findings. A CT scan of the abdomen and pelvis with contrast revealed that contrast material was leaking from the left kidney and surrounding tissues, indicating damage to the left renal structures. Additionally, there was also leakage of contrast material from the right ureter, which did not appear to be functioning properly. This led to a buildup of urine in the pelvis, a condition known as a pelvic urinoma, likely due to a tear in the right distal ureter (Figures 3-5). A urologist was consulted, who advised a CT urogram (Figure 6). It showed a left-sided forniceal rupture secondary to back pressure changes. The right distal ureter was not visualized, and contrast was seen pooling near the right distal ureter, leading to a urinoma suggestive of a right distal ureteral rupture.

Management

With CT urogram, diagnosis of left ureteric injury was confirmed. So, the initial plan for laparotomy was abandoned. Two units of packed red blood cells and two units of fresh frozen plasma were transfused. She had a right percutaneous nephrostomy with left ureteric antegrade double-J stenting and CT-guided drainage of pelvic collection. Double-J stent was removed after eight weeks. A week later, cystoscopy, right ureteroscopy, and antegrade pyelography were performed. Intraoperative findings of an obliterated right ureter at about 4 cm from the ureteric orifice with severe adhesions in the site of the right lower ureter were observed. A long ureteric stricture of about 8 cm was identified. Then, right ureteric reimplantation (Boari flap) + right double-j stent insertion were carried out. She had an uneventful post-operative period. She was discharged home in stable condition. The right nephrostomy tube was removed after one week. She had regular urology clinic follow-ups for six months.

## Discussion

The exact prevalence of ureteric injuries during pelvic surgery is unclear, but numerous studies suggest that the incidence ranges from 0.5% to 1.5% [1]. While less than 25% of these injuries are identified during surgery, they often go undetected and can lead to complications, such as urinary tract infections, ureterovaginal fistulas, and the formation of urinomas [1-3]. 

During open or laparoscopic surgery for benign or malignant urological conditions, there is a risk of acute complications. This risk is heightened by several factors, including advanced cancer, obesity, and the presence of adhesions from prior surgery, radiation, or inflammatory disease. Additionally, patients with a history of pelvic inflammatory disease, endometriosis, or broad ligament leiomyomas may also be more susceptible to complications. Furthermore, the skill level of the surgeon is also a critical factor, with low-volume surgeons who have performed fewer than 10 surgeries per year being at a higher risk of complications due to their limited experience [5].

The urinary bladder is the most commonly injured organ during surgery, affecting 60-70% of patients. The ureter is the next most frequently affected, with 24-30% of patients experiencing damage [5,6]. While bladder injuries are often detected and repaired during surgery, ureteric injuries, which are increasing due to the growing use of laparoscopic procedures, tend to be diagnosed later, typically between one week and one month after surgery [6-8]. In fact, almost two-thirds of ureteric injuries are diagnosed post-operatively. Notably, most ureteric injuries during hysterectomy occur at the pelvic brim near the infundibulo-pelvic ligament or along the pelvic side wall where the ureter crosses the uterine artery [9].

Iatrogenic ureteric injuries (IUIs) are most common after peripartum, laparoscopic, and abdominal radical hysterectomy. This is followed by IUI during robotic, laparoscopic, and abdominal hysterectomy for benign causes [5]. Of these injuries, 91% occur at the distal ureter. The most common location of ureteric injuries during hysterectomy is just lateral to the vagina, where the uterine artery crosses over it to enter the uterus. This accounts for approximately 65% of all cases. The remaining sites are less common, with 12% occurring at the infundibulopelvic ligament and 6% at the angle of the vaginal fornix [4,9,10]. Injury types vary, with 50% involving ligation (either partial or complete), 35% involving transection, and the remaining 15% involving excision of several centimeters of the ureter. The left and right ureters are equally affected by these injuries [10]. IUIs are most commonly caused by electrosurgery in nearly one-third of the cases, and the rest are caused during adhesiolysis and extensive dissection, transection with scissors, or during suturing [6].

Urinoma is a mass of encapsulated collection of urine due to continuous extravasation as a result of interruption in the urinary collection system producing internalized urine. It may occur spontaneously in the presence of obstruction after a closed renal or urinary injury or surgical operation. The post-traumatic leakage of urine is not infrequent (2-18%). The formation of a urinoma requires three essential conditions: a functioning kidney that is able to produce urine, a rupture or tear in the collecting system, and the presence of an obstruction in the ureter that prevents the urine from flowing out of the kidney properly. In the next two to five days, the extravasated urine causes lipolysis of the perirenal fat. It leads to walling off the collection by a fibrous capsule due to a fibroblastic reaction [4,11].

The uniqueness of this case lies in the initial wrong impression that the urinoma was hematoma when the collection was seen on ultrasound, as it appeared septated and echogenic due to infection. The patient, also being anemic (hemoglobin, 8.2 gm/dL) following hysterectomy, suggested concern about a bleeding vessel. She was also making a good amount of urine. The patient could have been taken for an unhelpful laparotomy. The patient’s condition might have undergone significant changes by the time they were referred to a urologist, potentially affecting the anatomy.

Definitive diagnosis is based on radiological evaluation. It aids in determining the cause, extent of the collection, and site of ureteric injury. Contrast material-enhanced CT with delayed imaging, CT cystography, and retrograde urethrography are the gold standard diagnostic imaging studies for diagnosing bladder and urethral injuries or conditions. Imaging-guided needle aspiration, antegrade and retrograde pyelography, and renal scintigraphy may have additional diagnostic roles [10-12]. 

Differential diagnoses of urinomas include ordinary ascites, abdominal or pelvic abscesses or hematomas, lymphoceles, cystic masses, or pancreatic pseudocysts. Seroma, bowel perforation, and enteric material leak are other differential diagnoses of post-operative fluid collection [13].

In cases where urine leaks require more extensive treatment, a combination of antibiotic therapy, minimally invasive procedures, and catheterization can be used to effectively manage the condition. This approach typically involves the placement of percutaneous urinoma drainage catheters, percutaneous nephrostomy catheters, and ureteral stents, as well as bladder drainage [8,9]. When implemented in the appropriate setting, this treatment plan can help reduce the risk of complications associated with urinoma and potentially avoid the need for surgical intervention. In our patient’s case, the result of CT imaging guided the need for further urological intervention in the form of Boari flap (right ureteric re-implantation) repair, as the right lower ureter was ruptured and developed stricture.

Small urinomas will usually reabsorb spontaneously and may not need drainage. Indications for intervention include sepsis irrespective of urinoma size, larger or increasing urinomas, and deranged renal function. Double-J stents may be satisfactorily used as the only treatment in cases with incomplete disruption of ureteral continuity. The use of a nephrostomy catheter alone may not lead to enough diversion of urine to allow spontaneous healing. Insertion of a nephrostomy catheter, in combination with insertion of a ureteral stent, in cases of persistent leakage from the collecting system is warranted [9].

IUI in distal ureters can be managed by ureteric reimplantation for injuries in the proximity of the bladder. The psoas hitch procedure is a surgical technique used to repair small defects in the distal ureters, particularly after a hysterectomy. It involves anchoring the ureter to the psoas muscle to create a tension-free and non-refluxing anastomosis, allowing for a secure and effective repair. Anterior bladder wall/Boari flap is used for reconstruction of defects that are wider and cannot be repaired by psoas hitch alone. Trans-uretero-ureterostomy is an alternative for the larger defect of the distal ureter or in cases where the bladder is small, fibrotic, and adherent, and bladder flaps are difficult to retrieve [9,10].

Prevention/recommendation

In cases with delayed post-operative complications, CT should be considered as the diagnostic modality of choice.

It is crucial to have a high level of suspicion for IUIs to ensure prompt recognition and repair, as delays can lead to complications, such as deep pelvic infections, fistula formation, and impaired kidney function.

While routine cystoscopy after gynecological surgeries is not universally recommended due to the added cost and time, it may be beneficial in cases where ureteric injuries are suspected.

Prophylactic ureteral stenting may be particularly useful in patients with a history of adhesions or previous pelvic surgery, as it can help mitigate potential complications.

This unusual case serves as a reminder to remain vigilant in identifying both intra-operative and post-operative urinary tract trauma, emphasizing the importance of timely diagnosis and repair to prevent adverse outcomes.

## Conclusions

Post-hysterectomy ureteric injury is not an uncommon complication of pelvic surgery. Simple hysterectomy for benign diseases is the most common cause of injury. Patients presenting with post-operative complications should be meticulously evaluated for all possible complications. In cases of suspected pelvic collections or ureteric injury, a CT scan remains the gold standard diagnostic modality. The patient with ureteric injury should be evaluated and intervened at the earliest. Correct forms of imaging can prevent unhelpful procedures and guide to appropriate intervention.
